# Expanding ocean protection and peace: a window for science diplomacy in the Gulf

**DOI:** 10.1098/rsos.230392

**Published:** 2023-09-27

**Authors:** Clare M. Fieseler, Nadia Al-Mudaffar Fawzi, Brian Helmuth, Alexandra Leitão, Mehsin Al Ainsi, Mohammad Al Mukaimi, Mohammad Al-Saidi, Fahad Al Senafi, Ivonne Bejarano, Radhouan Ben-Hamadou, Josh D'Addario, Ahmad Mujthaba Dheen Mohamed, Bruno W. Giraldes, Lyle Glowka, Maggie D. Johnson, Brett P. Lyons, Daniel Mateos-Molina, Christopher D. Marshall, Sayeed Mohammed, Pedro Range, Mohammad Reza Shokri, John M. K. Wong, Nicholas D. Pyenson

**Affiliations:** ^1^ National Museum of Natural History, Smithsonian Institution, Washington, DC, USA; ^2^ Marine Science Centre, University of Basra, Basra, Iraq; ^3^ Coastal Sustainability Institute and School of Public Policy and Urban Affairs, Northeastern University, Boston, MA, USA; ^4^ Environmental Science Center, Qatar University, Doha, Qatar; ^5^ Department of Biological and Environmental Sciences, College of Arts and Sciences, Qatar University, Doha, Qatar; ^6^ Center for Sustainable Development, College of Arts and Sciences, Qatar University, Doha, Qatar; ^7^ Marine Science Department, College of Science, Kuwait University, Kuwait City, Kuwait; ^8^ Department of Biology, Chemistry, and Environmental Sciences, American University of Sharjah, Sharjah, UAE; ^9^ The Open Data Institute, London, UK; ^10^ Biodiversity Strategies International, Abu Dhabi, UAE; ^11^ Red Sea Research Center, Biological and Environmental Sciences and Engineering Division, King Abdullah University of Science and Technology, Thuwal, Kingdom of Saudi Arabia; ^12^ NEOM Nature Reserve, NEOM, Tabuk, Saudi Arabia; ^13^ Emirates Nature - World Wide Fund for Nature, Dubai, UAE; ^14^ Department of Marine Biology, Texas A&M University, Galveston Campus, Galveston, TX, USA; ^15^ Department of Ecology and Conservation Biology, Texas A&M University, College Station, TX, USA; ^16^ Independent Researcher, Doha, Qatar; ^17^ Department of Animal Sciences and Marine Biology, Faculty of Life Science and Biotechnology, Shahid Beheshti University, G.C., Tehran, Iran; ^18^ Aquatic Research Center, Ministry of Environment and Climate Change, Doha, Qatar

**Keywords:** transboundary conservation, multicultural understanding, ocean governance, biodiversity, Arabian/Persian Gulf

## Abstract

The ecological state of the Persian or Arabian Gulf (hereafter ‘Gulf') is in sharp decline. Calls for comprehensive ecosystem-based management approaches and transboundary conservation have gone largely unanswered, despite mounting marine threats made worse by climate change. The region's long-standing political tensions add additional complexity, especially now as some Gulf countries will soon adopt ambitious goals to protect their marine environments as part of new global environmental commitments. The recent interest in global commitments comes at a time when diplomatic relations among all Gulf countries are improving. There is a window of opportunity for Gulf countries to meet global marine biodiversity conservation commitments, but only if scientists engage in peer-to-peer diplomacy to build trust, share knowledge and strategize marine conservation options across boundaries. The Gulf region needs more ocean diplomacy and coordination; just as critically, it needs actors at its science-policy interface to find better ways of adapting cooperative models to fit its unique marine environment, political context and culture. We propose a practical agenda for scientist-led diplomacy in the short term and lines of research from which to draw (e.g. co-production, knowledge exchange) to better design future science diplomacy practices and processes suited to the Gulf's setting.

## 1. Introduction

**Figure 1.**
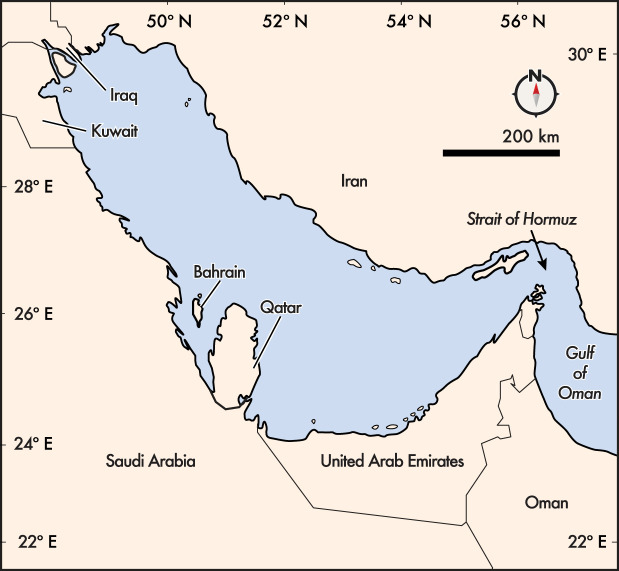
Map of the Gulf and the eight Member States of ROPME (Regional Organization for the Protection of the Marine Environment; table 1; map adapted from [2]). The Strait of Hormuz divides the inner ROPME Sea Area (i-RSA) to the west from the Middle RSA (M-RSA) to the east and Gulf of Oman; the Outer RSA (O-RSA) is not featured on this map.

The recent softening of geopolitical tensions among many Middle Eastern countries opens the possibility for increased cooperation on climate change and biodiversity loss that centres on the semi-enclosed 960 km-long Gulf^1^ figure 1 and table 1 [1]. Environmental challenges require the kind of government-to-government cooperation (e.g. dialogue) and coordination (i.e. higher-level cooperation) that other issues, such as public health, have newly received. The success of recent and anticipated commitments to legally binding ocean conservation targets depends on transnational coordination. For example, achieving the major aims of the Kunming-Montreal Global Biodiversity Framework (GBF) by 2030 will require transboundary data acquisition and sharing, as well as networking newly planned marine protected areas (MPAs) in the Gulf at a whole system level [3]. Considering emerging global frameworks, such as the UN Decade of Ocean Science, we argue that scientists working in the Gulf have an important window of opportunity to strengthen cooperation and build a foundation for state-led ocean diplomacy in the coming years.

**Table 1.** An alphabetized summary table of the organizations and entities for science diplomacy in the Gulf and surrounding the Arabian Peninsula mentioned in the main text.

organization abbreviation	full name	headquarters (if applicable)	founded	mission	member states (if applicable)
Emirates Nature - WWF	World Wide Fund for Nature (WWF) Regional Office	Dubai, United Arab Emirates	2001	As a regional organization in the Gulf, it generates scientific information and raises awareness to support science-based solutions and policies for addressing climate change and preserving the seas, land, and their interconnected biodiversity.	
GCC	Gulf Co-operation Council for the Arab States of the Gulf	Riyadh, Saudi Arabia	1981	A regional cooperation system whose objectives are: to effect coordination, integration and inter-connection between member states in all fields in order to achieve unity between them; to deepen and strengthen relations, links and areas of cooperation now prevailing between their peoples in various fields; to formulate similar regulations in various fields including economic and financial affairs, commerce, customs, communications, education and culture, social and health affairs, information and tourism, and legislative and administrative affairs; to stimulate scientific and technological progress in the fields of industry, mining, agriculture, water and animal resources; to establish scientific research; and to establish joint ventures and encourage cooperation by the private sector for the good of their peoples.	Bahrain, Kuwait, Oman, Qatar, Saudi Arabia, United Arab Emirates
ROPME	Regional Organization for the Protection of the Marine Environment	Kuwait City, Kuwait	1979	Coordinate efforts of the eight Member States towards protection of the marine and coastal environment and ecosystems in the ROPME Sea Area (RSA) against marine pollution and stressors that might be induced from developmental activities or/and other drivers of change.	Bahrain, Iran, Iraq, Kuwait, Oman, Qatar, Saudi Arabia, United Arab Emirates
TRSC	Transnational Red Sea Center	Lausanne, Switzerland	2019	A scientific research organization based at Ecole Polytechnique Fédérale in Lausanne with the support of the Swiss Confederation to raise awareness and promote dialogue to bridge science and diplomacy for the future of coral reefs, in the Red Sea and beyond.	Egypt, Eritrea, Israel, Jordan, Saudi Arabia, Sudan, Yemen
UNEP RSP	United Nations Environment Programme Regional Seas Programme	Nairobi, Kenya	1974	Implements region-specific activities, bringing together stakeholders including governments, scientific communities and civil societies.	The Regional Organization for the Protection of the Marine Environment (ROPME) Sea Area (RSA) falls under RSP's Non-UNEP administered Regional Seas Conventions and Action Plans (RSCAPs). This type of RSCAPs is under the auspices of UNEP, but another regional body (ROPME) provides the Secretariat and administrative functions.

There is no universal definition of ‘science diplomacy' nor a unifying conceptual theory. Classic case studies in science diplomacy—including the Antarctic Treaty (1959), the Outer Space Treaty (1967) and the creation of the Arctic Council (1996)—demonstrate how science engagement makes grander objectives possible (e.g. opening diplomatic channels, delimiting zones for peace, non-military forums for knowledge sharing). However, these examples of science diplomacy often reflect Western views of governance [4], conflict management [5] and foreign policy [6]. There is a need for multicultural understandings of science diplomacy processes, including the differential roles of actors. Just as peace scholars argue that peace and reconciliation processes must be redefined by each society [7], science diplomacy similarly requires updates at the science-society interface. Here, we identify common environmental threats to neighbouring Gulf countries and the growing alignment of their scientific priorities and environmental values, despite persisting ideological tensions. Crucially, our agenda for Gulf scientists and their international collaborators focuses not on politicized values, but rather on common values and interests about the marine environment that are starting points to develop new forms of science diplomacy across borders. We favour the term ‘reconciliation' for the transboundary work needed for the Gulf's future.

## 2. Recognizing common challenges

As a restricted marine basin, the Gulf's maritime political boundaries are crowded among its neighbouring states of Bahrain, Iran, Iraq, Kuwait, Oman, Qatar, Saudi Arabia and the United Arab Emirates. During the summer, the Gulf is the world's hottest coastal environment. Many of its species live close to their physiological limits [8,9]. Climate-induced changes to seawater temperature (greater than 35°C), salinity (greater than 40 psu), the frequency and strength of shamal winds, dust storms and cyclones [10], and a decrease in pH and dissolved oxygen all make the Gulf a laboratory for the most acute effects of future climate change [8,11,12].

The Gulf is also a global hotspot for rapid coastal development. Coastal reclamation, desalination plants and infrastructure projects are causing further declines in the Gulf's marine ecosystems (e.g. coral reefs, mangroves, mudflats, seagrass habitats) [13]. Biodiversity losses are associated with habitat destruction, fishing and climate change, meanwhile sewage discharges, brine disposal, maritime transportation and oil production activities, all pose acute localized threats. The expansion of the petroleum industry is a major contributor to marine pollution in the Gulf, creating the constant threat of oil spills, both large and small, which can expand across boundaries.

Responding to these common challenges across the region requires an integrative understanding of gaps in risk mitigation, hazard management and the magnitude of restoration measures needed to sustain marine species [14]. Regional habitat mapping, especially in offshore areas [15], could better pinpoint the status, trends and recovery capacity of habitats along with the sensitivity of migratory species and ecosystem engineers, such as dugongs. There is high marine endemism in some Gulf marine environments, and the trophic cascades that may arise from endemic species' disappearances are not well understood [16].

Previous calls for regional collaboration on ecosystem-based management (EBM) solutions have included coordinated ecosystem monitoring, transboundary fisheries governance, and a singular integrated conservation network of MPAs with other area-based conservation measures [17–19]. There is strong evidence that transboundary cooperation provides a structure for solutions especially when large-scale climate change has variable and tele-connected consequences with local drivers (e.g. land use). For example, at the head of the Gulf, Iraq's severe water shortages can change the salinity, temperature, sediment supply and pollutants of the entire Gulf [20]. Marine spatial planning with EBM approaches could be an effective means of addressing marine biodiversity and the needs of diverse Gulf stakeholders [21], but only a few Gulf MPAs have marine spatial management plans that accommodate best practices, like zoning for diverse uses. One assessment of MPA management found low overall effectiveness (34%) across the entire Gulf [19].

## 3. Taking stock of environmental cooperation

The recent integration of regional cooperation on Gulf marine conservation began in the 1970s. Using the Mediterranean Action Plan as a template, the Regional Organisation for the Protection of the Marine Environment (ROPME) formed in 1978 among all Gulf coastal nations and marked early successes in controlling marine pollution. ROPME is affiliated with UNEP's Regional Seas Programme (RSP). Although widely seen as achievements of environmental diplomacy in the twentieth century, many of the 18 RSPs affiliated with UNEP today, including ROPME, are challenged by weak compliance and geopolitical issues [22].

Intergovernmental organizations and global non-governmental organizations (NGOs) have also facilitated environmental diplomacy. For example, the World Wide Fund for Nature (WWF) regional office in UAE has co-led Gulf coral reef conservation planning across the coastlines of Abu Dhabi and Qatar [23]. Gulf countries, including the notable recent addition of Iraq, have joined UN multilateral environmental conventions, which facilitate information sharing, social encounters and trust building. Countries belonging to the Gulf Cooperation Council (GCC) have had a long-standing obligation to cooperate under the GCC Convention on the Conservation of Wildlife and their Natural Habitats (2001) and the Convention's wildlife committee meets on a regular basis to coordinate implementation of the Convention. They have also become more engaged with the global sustainable development agenda and, subsequently, green growth networks, which has raised the visibility of energy transition and climate change within the GCC [24]. While traditional science diplomacy continues to spark coordination on global climate action, the GCC alone cannot offer inclusive ocean diplomacy across the Gulf because Iraq and Iran are not GCC member states.

There is an urgent need to develop an inclusive framework to address climate-adjacent environmental challenges, such as marine biodiversity decline. Recent ROPME-led convenings on climate change with scientists and political leaders [25] demonstrate a regional capacity to have dialogue on critical issues. These efforts need to be dramatically expanded and enhanced to mitigate multiple threats to marine biodiversity in the Gulf that have not yet been addressed comprehensively. GCC countries have strong financial capabilities, governing coherence and legacies of cooperation to facilitate trust building with the Gulf's two non-GCC countries. Efforts to accelerate the mitigation of marine biodiversity loss, especially for regional agenda-setting, need to include scientists from all Gulf nations as a way to ease the perception of representative bias; also, scientists have social networks, credibility and portable expertise [26], all of which lends further weight to transboundary cooperation that can be elevated and useful to intergovernmental dialogue. Although Gulf countries may have different environmental agendas, science diplomacy research shows that ‘productive tension' [27] can lead to common ground where consensus is not a necessary objective. Productive tensions exist among participants that have different perspectives, values and ways of knowing; the product, for science diplomacy, arises from co-production at the boundaries of issues and discussion [27]. Such a model fosters continuous dialogues, trust building and data sharing among scientists.

Data sharing requires the stewardship of data for public benefit and building strong infrastructure, like data institutions. In the Gulf, ROPME could meet this institutional need as a multilateral organization. Another model for data diplomacy involves fostering neutral boundary organizations. The Transnational Red Sea Center (TRSC) demonstrates this approach on the western side of the Arabian Peninsula. The TRSC facilitates knowledge sharing about coral reef habitats across the eight nations bordering the Red Sea. It seeks to overcome historical, conflict driven isolation through the diplomatic neutrality of the Swiss Foreign Ministry, which plays a boundary-spanning role by funding and advising the centre, including its transboundary expeditions, with the goal of cooperative coral science and dialogue [28]. We see promise in TRSC's innovative design, built around a capable boundary-spanning institution with expertise in knowledge exchange.

## 4. Building towards ocean diplomacy

Science diplomacy builds common interests among allies and historical adversaries. But even flagship settings for science diplomacy (e.g. Arctic, Antarctica) show that trust and scientist-diplomat engagement can decline or fall to geopolitics [4,5]. Scientists and their peer networks have a role to play in bolstering trust. And, with existing boundary organizations willing to facilitate or expand existing networks, scientists do not need to wait on formal diplomatic channels to act.

First, regional funding for scientists interested in networked Gulf studies should be dramatically expanded. Currently, such funding is insufficient to meet the moment and nationally delimited; pan-Gulf integration is often accidental. The GCC has strong funding capabilities and explicit coordinating infrastructure. Also, many national-level funding programs could provide enhanced regional cooperation support (e.g. Kuwait Foundation for the Advancement of Sciences (KFAS), Qatar Foundation's Qatar National Research Fund (QNRF), Iran National Science Foundation (INSF)). Additional financing can come from foundations, the private sector and international organizations.

Second, peer networks among scientists should be strengthened to increase trust through data sharing. Conservation planning aimed at geopolitically transboundary species, such as commercial fish species, sea turtles and marine mammals, represents a unique area for sharing data. Grassroots networks among scientists are already building regional capacity for sharing data with global networks, as demonstrated by the ongoing engagement between the Arabian Seas Whale Network and the International Union for Conservation of Nature's Important Marine Mammal Areas process [29]. ROPME can also catalyse scientist-led, peer-to-peer data sharing networks by promoting data acquisition and policies that address marine biodiversity challenges. Open access (OA) policies can also improve data sharing for government-supported research funding, as KFAS has mandated. By moving networks from closed to OA practices, scientists can provide avenues for bottom-up initiatives, such as the new creation of a much-needed Gulf-wide marine mammal stranding network, which can later be formalized through top-down diplomatic action.

Third, scientists in the Gulf can lead transparent, participatory and systematic approaches to area-based conservation measures, which will accelerate progress towards global biodiversity conservation targets. Creating a comprehensive and ecologically coherent MPA network will provide an EBM strategy for enhancing the resilience of regional biodiversity in the face of climate change. We recommend that scientists, alongside capable boundary spanning organizations, help co-develop coordinated management metrics across MPAs to ensure effective conservation and connectivity [3]. The Gulf currently has no large transboundary MPAs that straddle maritime boundaries (i.e. marine peace parks). While peace parks require shared governmental maintenance for long-term viability [30], their establishment could solve conservation disconnect. The Gulf of Salwa, for example, harbours thriving dugong populations and expansive intact seagrass beds that bridge the territorial waters of Bahrain, Qatar and Saudi Arabia [31,32]. We recommend this site for the region's first transboundary marine park.

One of the problems with studies on marine ecosystems in the Gulf is the lack of integration in research topics. This disconnection is partially attributable to a lack of knowledge about ongoing research projects in the regional countries that suffer from journal paywalls and publication fees especially for conservation and biodiversity publications. To resolve this problem, we advocate creating a project database as a first step, which can be established and managed by the ROPME. A second solution is to create a forum such as Coral-List, which provides a forum for online discussion and announcements pertaining to coral reef ecosystem research, conservation and education. Having a forum for the researchers in the Gulf may help define integrative research topics that bridge the gap of information on marine ecosystems in the region. Identifying gaps will be especially useful for planning and coordination across all GCC member states and other pan-Gulf transboundary organizations. For example, the establishment of a network of Gulf MPAs requires understanding fundamental biological processes (e.g. coral larvae exchange) among a large number of marine ecosystems across all member states.

Scholars have long held-up less populated regions, like the Arctic, as settings for studying and advancing science diplomacy, while heavily populated and politically complex regions, like the Gulf, have been overlooked [1]. We argue that non-pristine and urbanizing regions should be considered for peer-to-peer diplomacy—also called Track 2 diplomacy—which the UN Decade of Ocean Science strives to spark as an organizing platform. This UN campaign asks scientists to better coordinate science across borders in the pursuit of the UN Sustainable Development Goals and ‘change how they organize themselves' [33]. In Gulf countries, there is no clear sense for how non-state actors (e.g. scientists, environmental NGOs) can engage with state actors (e.g. ministerial-level politicians, diplomats), which is closer to traditional Track 1 diplomacy. Governments that can build bridges across Track 1 and Track 2 diplomacy may garner more public awareness and support for their environmental agendas. For example, recent surveys indicate that Qatar residents view scientists as the most trustworthy sources of environmental information, eclipsing government sources by nearly twofold [34]. Diplomacy-oriented scientists and science-grounded diplomats can co-produce creative forms of diplomacy that fit the region's culture and ongoing reconciliation.

In addition to increased diplomacy in the region, we call for better practices and processes for ocean diplomacy. This must be informed by relevant lines of social science scholarship—for example, the field of knowledge co-production, which captures the body of scholarship organized around ‘science-policy interface(s)' and other related concepts. Co-production has deepened scholars' and practitioners' understanding of the role of trust and boundary spanning organizations in informing policy-making [35–37]. Multiple case studies from the co-production literature support the claim that successful collaborations and cooperative networks at science-policy interfaces are underpinned by trust-building processes [38,39]. We propose that co-production scholarship may be used to build models for networked Gulf scientists to increase trust and build social capital when conducting pan-Gulf studies. The Gulf should also be a site for new co-production and governance scholarship to expand our understanding of understudied themes at the science-policy interface, like consensus. In contrast to most claims made by co-production scholars [40,41], consensus may not always be more effective than non-consensus at informing environmental policy or governance. In fact, the Gulf region may be a critical transboundary setting for studying models of useful scientific engagement, credibility and cooperation in the absence of consensus and agreement.

The declining Gulf marine environments and the aligning of environmental interests, coupled with renewed regional diplomacy efforts, create an unusual convergence of drivers to begin understanding new modes of engagement. This window of opportunity for Gulf scientists to voluntarily integrate processes, data and strategize conservation research is potentially narrow, or at least time-sensitive relative to climate goals. Gulf governments, especially those states committing to ambitious 30 × 30 conservation targets [42], can take advantage of existing peer networks among scientists as a template for intergovernmental research support, trust building and strategic planning for ocean protection, as is being done elsewhere [43]. This article is one example of the willingness of researchers, working in the Gulf and at other international institutions, to set aside differences and cooperate over our common ambitions to protect marine biodiversity.

## Data Availability

This article has no additional data.

## Footnotes

1 The name of the water body at the centre of this discussion remains contentious. The one-word name ‘Gulf’ is used here, as previous publications have done (e.g. [1]), to omit any geographic descriptors that detract from a productive dialogue. ‘Inner Gulf of the ROPME Sea’ or ‘ROPME Sea Area’ are names accepted by all coastal states of the Gulf via the ROPME Council (see also figure 1 and table 1). ‘Persian Gulf’ and ‘Arabian Gulf’ are other names used by states.
